# Imaging diagnosis and differential diagnosis of extraskeletal osteosarcoma

**DOI:** 10.1186/s12885-023-11731-3

**Published:** 2024-01-02

**Authors:** Xiao-chun Wang, Ling Zhang, Jiong-bin Lin, Xiao-yao Huang, Jing-hong Liang, Jian-ping Zhong, Ji-dong Peng, Jun-yuan Zhong

**Affiliations:** 1 Ganzhou Institute of Medical Imaging, Ganzhou Key Laboratory of Medical Imaging and Artificial Intelligence, Medical Imaging Center, Ganzhou People’s Hospital, The Affiliated Ganzhou Hospital of Nanchang University, Ganzhou Hospital-Nanfang Hospital, Southern Medical University, Ganzhou, 341000 China; 2 Department of Radiology, Nanfang Hospital, Southern Medical University, Guangzhou, 510515 China

**Keywords:** Extraskeletal osteosarcoma, CT, MRI

## Abstract

**Objective:**

The aim of this study was to investigate the clinical, imaging and pathological features of extraskeletal osteosarcoma (EOS) and to improve the understanding of this disease and other similar lesions.

**Methods:**

The data for 11 patients with pathologically confirmed extraosseous osteosarcoma, including tumour site and size and imaging and clinical manifestations, were analysed retrospectively.

**Results:**

Six patients were male (60%), and 5 were female (40%); patient age ranged from 23 to 76 years (average age 47.1 years). Among the 11 patients, 7 had clear calcifications or ossification with different morphologies, and 2 patients showed a massive mature bone tumour. MRI showed a mixed-signal mass with slightly longer T1 and T2 signals in the tumour parenchyma. Enhanced CT and MRI scans showed enhancement in the parenchyma. Ten patients had different degrees of necrosis and cystic degeneration in the mass, 2 of whom were complicated with haemorrhage, and MRI showed “fluid‒fluid level” signs. Of the 11 patients, five patients survived after surgery, and no obvious recurrence or metastasis was found on imaging examination. One patient died of lung metastasis after surgery, and 2 patients with open biopsy died of disease progression. One patient died of respiratory failure 2 months after operation. 2 patients had positive surgical margins, and 1 had lung metastasis 6 months after operation and died 19 months after operation. Another patient had recurrence 2 months after surgery.

**Conclusion:**

The diagnosis of EOS requires a combination of clinical, imaging and histological examinations. Cystic degeneration and necrosis; mineralization is common, especially thick and lumpy mineralization. Extended resection is still the first choice for localized lesions. For patients with positive surgical margins or metastases, adjuvant chemoradiotherapy is needed.

## Background

Extraskeletal osteosarcoma (EOS) or soft tissue osteosarcoma refers to osteosarcoma that occurs within the soft tissue and does not originate from bone. EOS is extremely rare, accounting for approximately 1% of all soft tissue sarcomas and approximately 4% of all osteosarcomas [1]. EOS has the same histomorphological characteristics as osteosarcoma that occurs in bone, but its biological behaviour and clinical manifestations are different from those of intraosseous osteosarcoma [2–4]. At present, the treatment for EOS mainly involves surgical resection and adjuvant chemoradiotherapy; despite treatment, EOS has a high rate of metastasis and recurrence, with a poor prognosis [1]. There are few studies on the imaging manifestations of EOS, and most are case reports. The imaging features of the disease are poorly understood by radiologists and clinicians, and the accuracy of preoperative imaging diagnosis is not high [5–7]. Diagnosis is usually made by needle biopsy or open biopsy. In this study, the clinical and imaging data for 11 patients with pathologically confirmed EOS were retrospectively analysed, and the data were compared with results reported in the literature to improve the understanding of the clinical and imaging signs of the disease and assist clinicians in determining surgical plans, treatment regimens and follow-up management.

## Methods

### Clinical data

The data for 11 patients with pathologically confirmed EOS from January 2008 to March 2023 were collected. The data mainly included clinical manifestations, imaging examination results, and pathology results. Eight patients underwent computed tomography (CT) examinations, and 5 of these patients also underwent magnetic resonance imaging (MRI) examinations; 1 patient underwent digital radiography (DR) and MRI examinations, 1 patient underwent ultrasound and DR examinations, and 1 patient underwent an MRI examination only. Four patients underwent enhanced CT examinations, and 6 patients underwent enhanced MRI examinations.

### CT and MRI examinations

At one hospital, CT was performed with a GE Revolution CT scanner using the following parameters: slice thickness, 5 mm; slice distance, 5 mm; tube voltage, 120 kV; and tube current, 382 mA. Multiplanar reconstruction (MPR) images were captured on the workstation. Iohexol (350 mg I/ml), a nonionic contrast agent, was used for contrast-enhanced CT scans at a dose of 1.2 ml/kg and a flow rate of 3.0 ml/s. A Siemens Verio 3.0T was employed for MRI using a fast spin echo sequence and the following imaging parameters: axial T1WI – TR/TE, 600/23 ms; T2WI – TR/TE, 4000/76 ms; slice thickness, 6 mm; and FOV 64 cm×64 cm. The contrast agent used for enhanced MRI was Gd-DTPA at a dose of 0.1 mmol/kg. After the contrast agent was injected intravenously, the site was scanned using the TSE sequence. The scanning parameters were as follows: axial T1WI – TR/TE, 640/23 ms; slice thickness, 6 mm; and FOV, 64 cm×64 cm; coronal T1WI – TR/TE, 653/20 ms; slice thickness, 4 mm; and FOV, 37 cm×44 cm; and sagittal T1WI – TR/TE, 650/20 ms; slice thickness, 4 mm; and FOV, 44 cm×37 cm.

At the other hospital, CT was performed with a Siemens SOMATOM Definition dual-source CT scanner using the following parameters: slice thickness, 5 mm; slice spacing, 5 mm; tube voltage, 120 KV; and tube current, 526 mA. MPR images were captured on the workstation. MRI plain and enhanced scans conducted with a 3.0 T superconducting MRI, GE Signal Excite, and corresponding coils were selected based on the examination site. Spin‒echo pulse sequences were used for all patients. The imaging parameters were as follows: axial T1WI – TR/TE, 4600/8.2 ms; T2WI – TR/TE, 4000/142.5 ms; slice thickness, 5 mm; NEX, 2; and FOV, 38 cm×38 cm. The contrast agent was Gd-DTPA, used at a dose of 0.1 mmol/kg and administered by a bolus injection into the cubital vein. After the contrast agent was injected intravenously, all parts were scanned using the FSE sequence. The scanning parameters were as follows: axial T1WI – TR/TE, 560/8.0 ms; slice thickness, 6 mm; and FOV, 38 cm×38 cm; coronal T1WI – TR/TE, 560/8.2 ms; slice thickness, 5 mm; and FOV, 40 cm×40 cm; sagittal T1WI – TR/TE, 550/8.5 ms; slice thickness, 5 mm; and FOV 40 cm×40 cm.

### Image analysis

The location, shape, size, density, and calcification of lesions were recorded by two senior radiologists. Morphology, boundary, signal, enhancement performance, necrotic cystic degeneration and haemorrhage on each MRI sequence were recorded. The results were compared with the histopathological findings.

## Results

### Image performance

In this study, the data for 11 patients with pathologically confirmed EOS were analysed; 6 patients were male (60%), and 5 were female (40%); patient age ranged from 23 to 76 years (average age 47.1 years). In 2 patients, EOS was located in the thigh (the space between the posterior muscles of the lower thigh and the space between the lateral muscles of the lower thigh); in 2 patients, EOS was located in the soft tissue of the buttocks (left buttocks and right buttocks); in 1 patient, EOS was located in the upper arm soft tissue; in 3 patients, EOS was located in the abdominal cavity (1 case in the lesser omentum sac, 1 in the uterus, and 1 in the ileocecal area); and for 1 patient each, EOS was located in the left lung, in the lower back, and in the chest wall. Soft tissue masses were observed in all 11 patients, with a maximum diameter ranging from approximately 3.0-16.2 cm and an average diameter of approximately 8.6 cm. Multiple masses were observed in 2 patients; the masses were located on the lateral side of the right thigh and the uterus; the remaining 9 patients had localized masses. The imaging data for the 11 patients are provided in Tables 1 and 2.

**Table 1:** CT findings of extraosseous osteosarcoma

Case	Gender	Age	Parts	DR/CT	CT enhancement	Calcification/ossification	Calcification shape
Case1	male	23	thigh	Invisible	/	None	/
Case2	male	27	thigh	Iso + slightly higher density	/	None	/
Case3	male	76	buttocks	isodensity	/	None	/
Case4	female	43	buttocks	/	/	None	/
Case5	male	56	Bursae omentalis minor	Iso + low + high density	Gradual enhancement is obvious	Yes	Patchy
Case6	female	59	Uterus	High density	Gradual enhancement is obvious	Yes	Clumps
Casse7	female	55	Abdominal cavity	Iso + low + high density	Progressive and moderate enhancement	Yes	Patchy
Case8	male	62	chest wall	Slightly lower + high-density	Mild fortification	Yes	Arc
Case9	female	36	upper arm	/	/	Yes	Patchy
Case10	male	42	Lower back	High density	/	Yes	Clumps
Case11	female	40	lung	Iso + slightly lower density	/	None	/

/:unchecked

**Table 2:** MRI findings of extraosseous osteosarcoma

Case	T1WI	T2WI	T1 enhancement	Cystic degeneration/necrosis	Fiber dividers	Envelope	Peripheral invasion
Case1	isosignal	Wait/high signal	Uneven noticeable intensification	Yes	None	Yes	Invasion of the common peroneal nerve
Case2	Low/equal/high signal	Low/equal/high signal	Uneven noticeable intensification	Yes	Yes	Yes	None
Case3	Low/high signal	Low/high signal	Uneven noticeable intensification	Yes	Yes	Yes	/
Case4	isosignal	High signal	Uneven noticeable intensification	Yes	Yes	None	/
Case5	/	/	/	Yes	None	/	Invading the stomach wall
Case6	Wait/low signal	Low/equal/high signal	Sustained significant reinforcement	Yes	None	None	Invasion of bladder and rectum
Case7	Wait/low/high signal	Low/high signal	Sustained significant reinforcement	Yes	None	Yes	Intestinal invasion
Case8	/	/	/	Yes	/	Yes	None
Case9	/	/	/	Yes	None	Yes	None
Case10	Equal/low signal	Low/equal/high signal	/	Yes	None	Yes	None
Case11	/	/	/	Yes	None	/	/

/:unchecked

Laboratory tests showed that serum alkaline phosphatase was elevated in 4 patients before treatment, with the highest level being 615 U (normal reference value 45–125 U/L), and normal in 4 patients before treatment; alkaline phosphatase was not monitored in 3 patients before treatment. Alkaline phosphatase levels were decreased in 2 patients after surgery or chemotherapy but were still higher than normal (227 U/L, 158 U/L). In one patient, the level decreased to within the normal range after treatment. Two of the 3 patients with normal alkaline phosphatase levels exhibited slightly increased levels (135 U/L, 128 U/L) after treatment. Lactate dehydrogenase (LDH) was elevated in 2 patients before treatment (284 U/L, 270 U/L (120–250 U/L)), and 2 patients had normal levels. One patient exhibited a decrease in the LDH level to within the normal range after treatment. Among the 11 patients, CA125 was increased (331.66 U/L, normal range 0–35 U/ml) in 1 patient, AFP was increased (8.64 ng/ml, 0–7.0 ng/ml) in 1 patient, and the other tumour markers were normal (CEA, AFP, CA125, CA199, etc.).

**Fig. 1:**
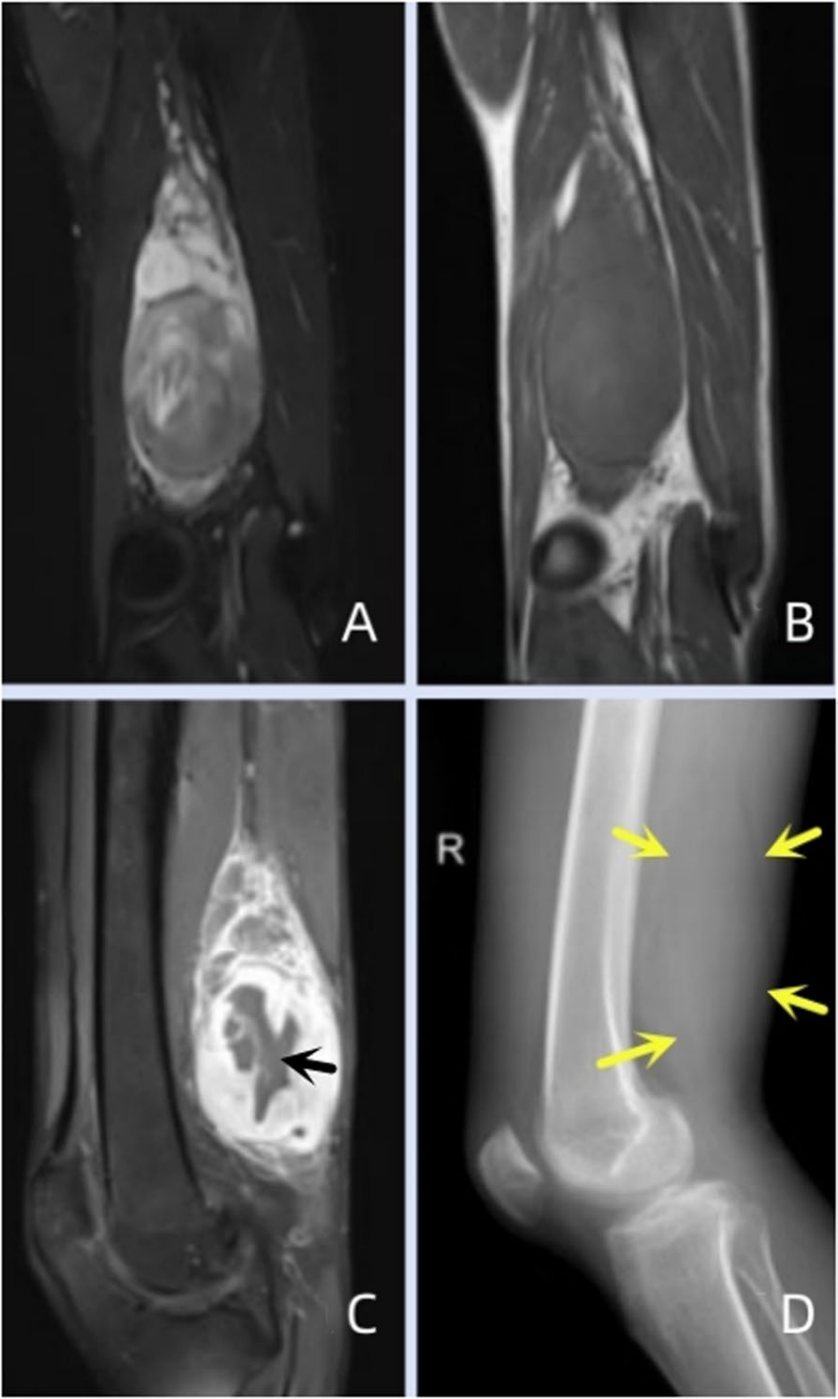
A 23-year-old man with EOS in the right thigh. **A**, **B **MR plain scan showing a mass with clear borders behind the femur in the lower thigh. The T1WI signal was similar to the muscle signal, and the T2WI signal was mixed. The parenchymal component had a slightly high signal, and the cystic part had a significantly high signal. **C **Enhanced MR scan showing progressively marked enhancement of the parenchymal component without enhancement in the cystic area (black arrow). **D **X-ray showing a mass resembling a muscle density mass (yellow arrow) without obvious calcification or ossification

**Fig. 2:**
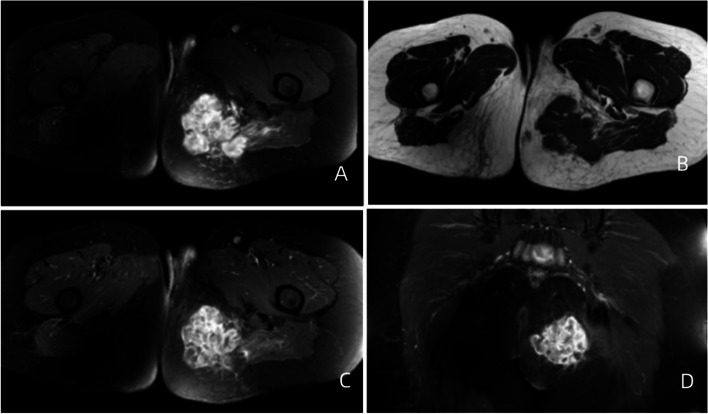
A 43-year-old woman with EOS in the left hip. A, **B **MR plain scan showing an irregular subcutaneous fat mass on the left buttock, lobulated, with high signal intensity on T2WI and isointensity on T1WI.  **C**, **D **Enhanced MR scan showing less uniform and significant enhancement

**Fig. 3:**
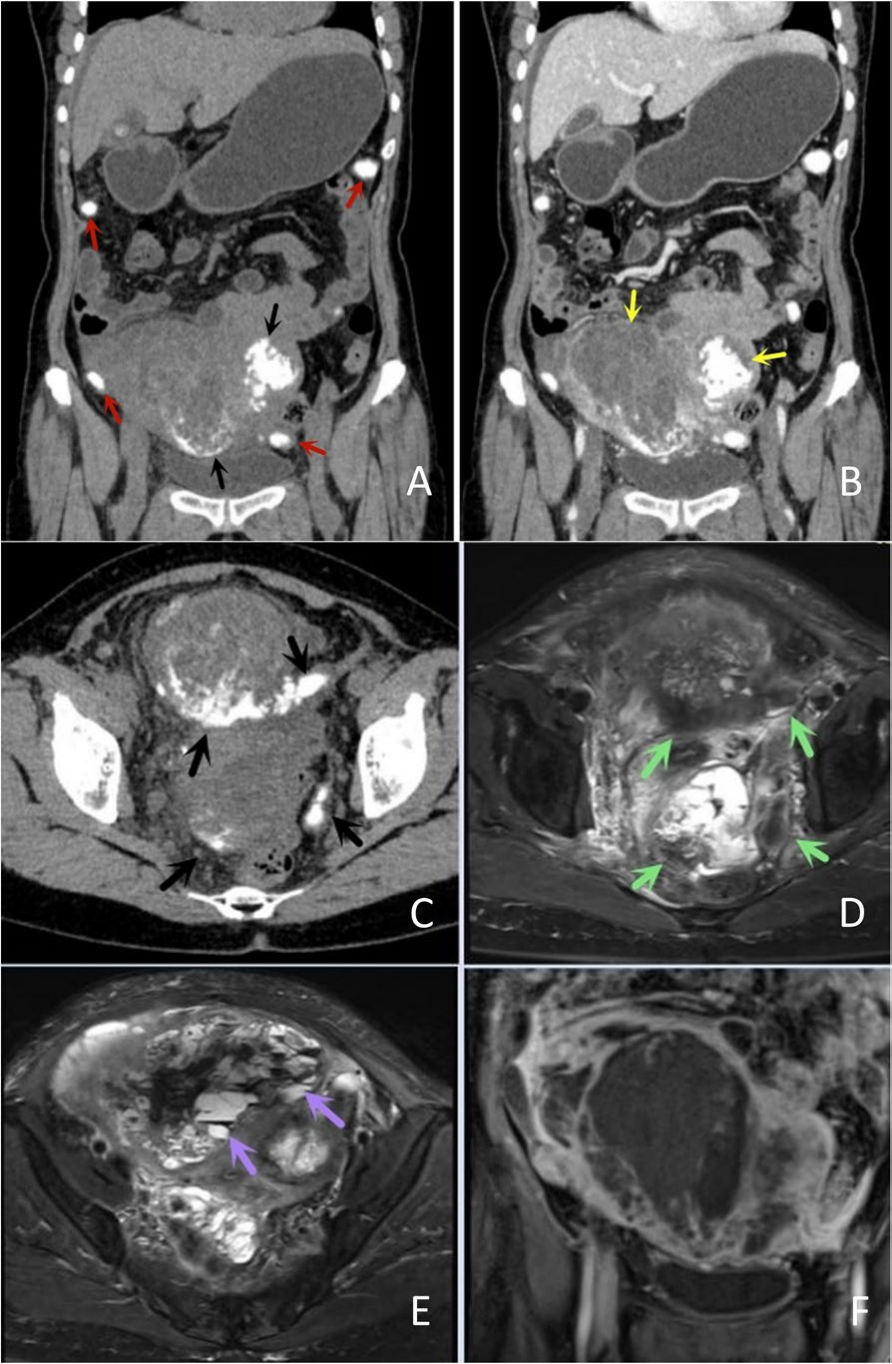
A 59-year-old woman with EOS in the uterus. **A**, **B **Coronal CT showing a large uterine mass with large, massive calcification or ossification (black arrow) and extensive cystic degeneration and necrosis (yellow arrow). Metastatic lesions with multiple calcifications in the abdominal cavity were also observed (red arrows). **C**, **D **Calcification or ossification was hypointense on MR imaging (green arrow).  **F **Mixed signals on T2WI, haemorrhage within the mass, and a fluid‒fluid level (purple arrow) were observed. **B**, **F **The enhanced CT and MR findings were consistent, showing less uniform and significant enhancement

**Fig. 4:**
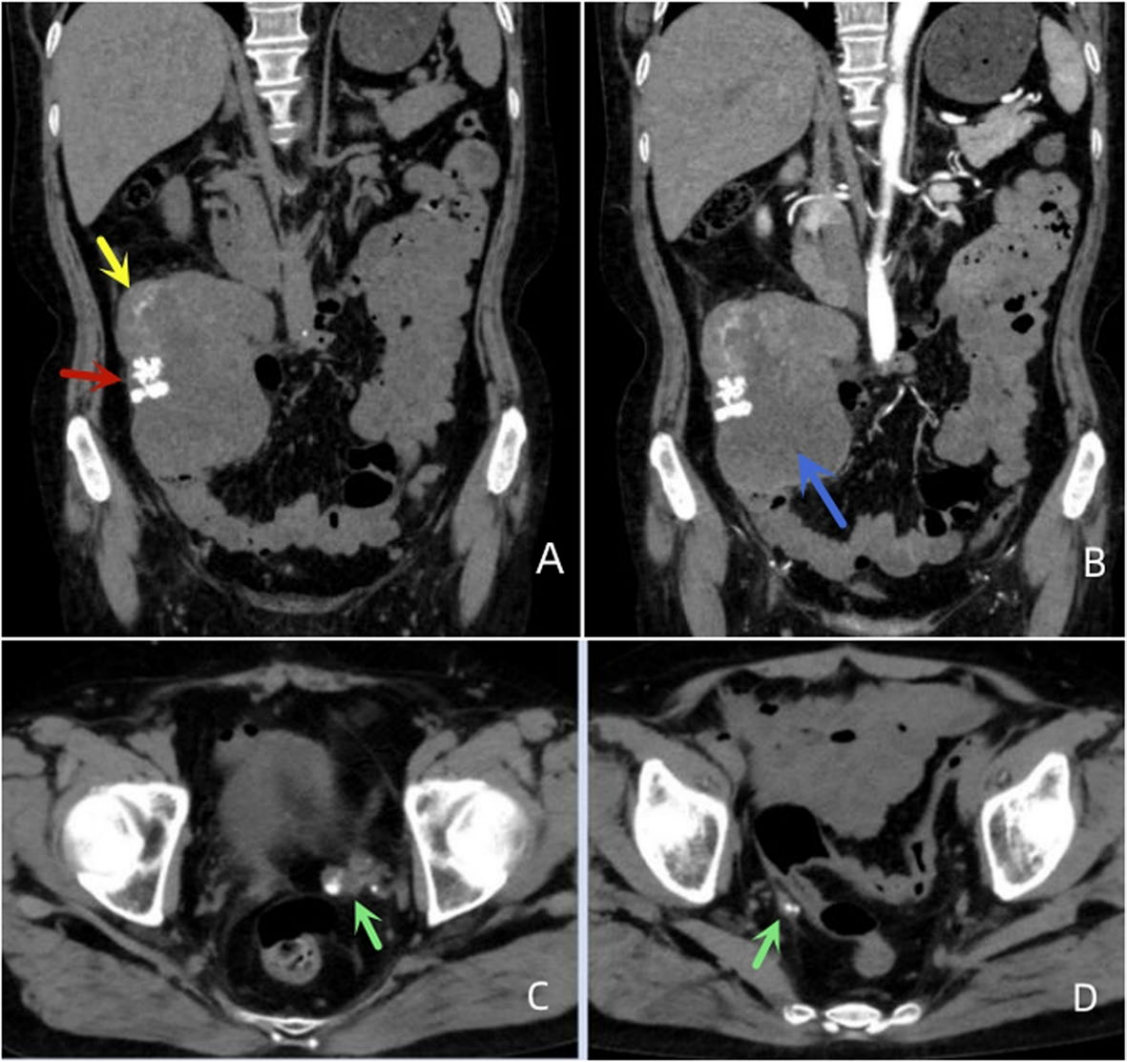
A 55-year-old woman with EOS in the abdominal cavity. **A **The mass was irregular in shape, with patchy, coarse-grained calcification or ossification within the mass on CT (red and yellow arrows). **B **Enhanced scan showing cystic/necrotic areas (blue arrows). **C**,** D **Pelvic metastases were found (green arrows)

**Fig. 5:**
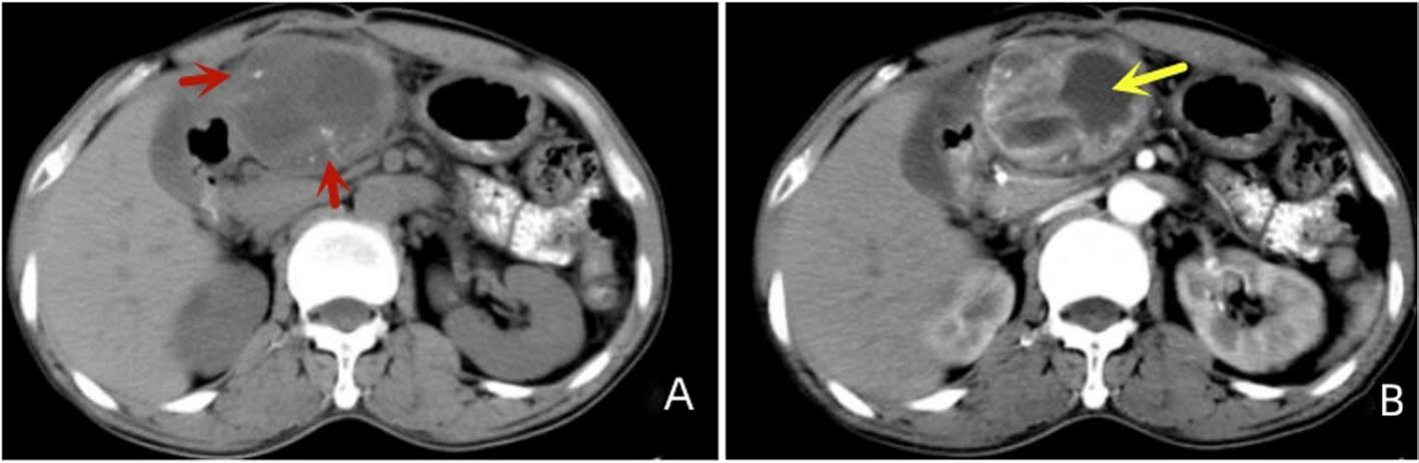
A 56-year-old man with EOS in the lesser omental bursa.  **A**, **B **CT showing a mass in the lesser omental cyst area, with coarse-grained calcification/ossification (red arrow) and cystic degeneration (yellow arrow); the mass was close relative to the gastric antral wall

**Fig. 6:**
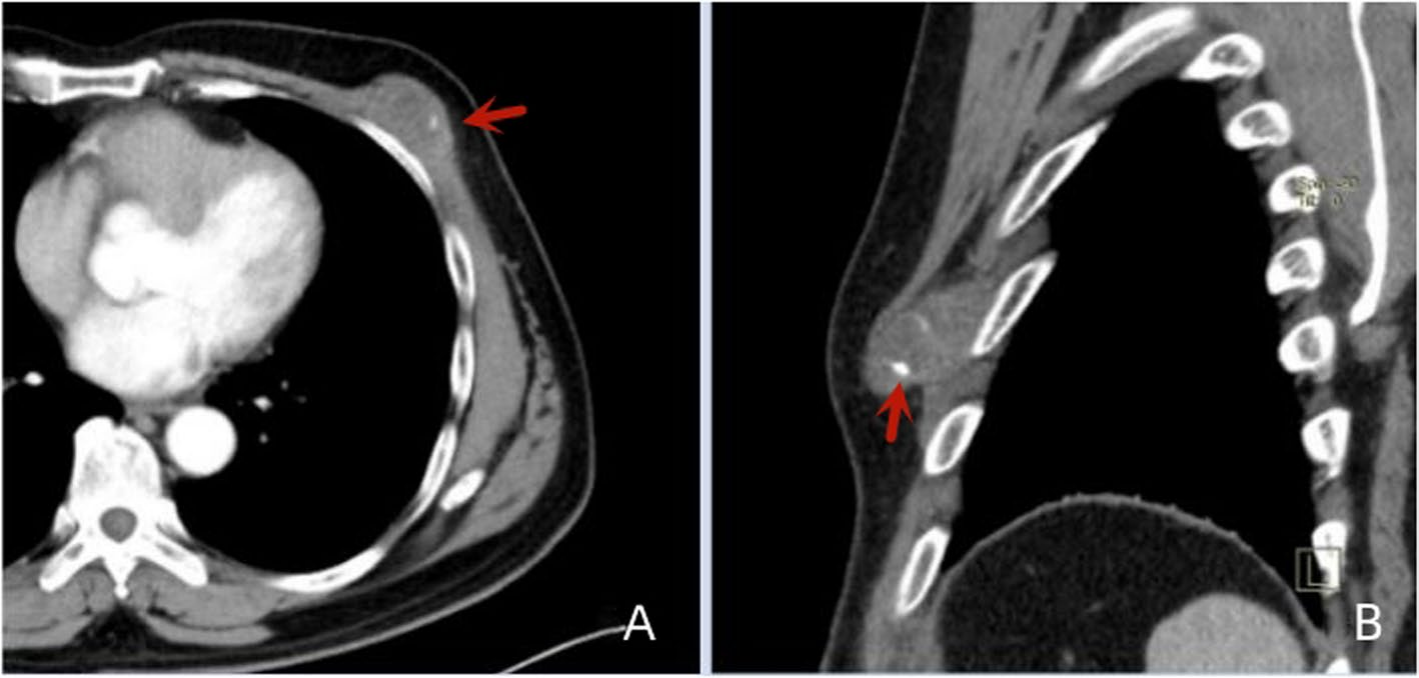
A 62-year-old man with EOS in the left chest wall. **A**, **B **Punctate calcifications (red arrows) at the edge of the CT plain scan and mild enhancement of the mass on an enhanced scan

Of the 11 patients, 7 had clear calcifications or ossification (Figs. 3, 4, 5 and 6), ranging from coarse granular to lumpy, and 2 had thick and massive mature tumours (Figs. 3 and 4), i.e., EOS of the ileocecum and uterus. Calcification or ossification was distributed centrally to the periphery of the mass, with others that were peripherally distributed. Ten patients had different degrees of necrosis and cystic degeneration in the mass (Figs. 1, 3, 4, 5 and 6), and 1 patient had no clear cystic degeneration on DR and ultrasonography; for 2 patients, “fluid‒fluid level” signs were observed on T2WI MRI (Fig. 3). The upper layer had a high signal, and the lower layer had a low signal (Table 2). Enhanced CT and MRI scans showed enhancement in the parenchyma and septa (Figs. 1, 2, 3, 4, 5 and 6). For six patients, there was less uniform and obvious enhancement, and for 1 patient, there was mild enhancement (Fig. 6). Fibrous septa were observed in 3 patients, with low signals on T1WI and T2WI. Capsules were present in 6 patients and were either intact or incomplete. In 4 patients, adjacent tissue invasion was observed, including in the nerves, bladder, and bowel (Fig. 5).

**Fig. 7:**
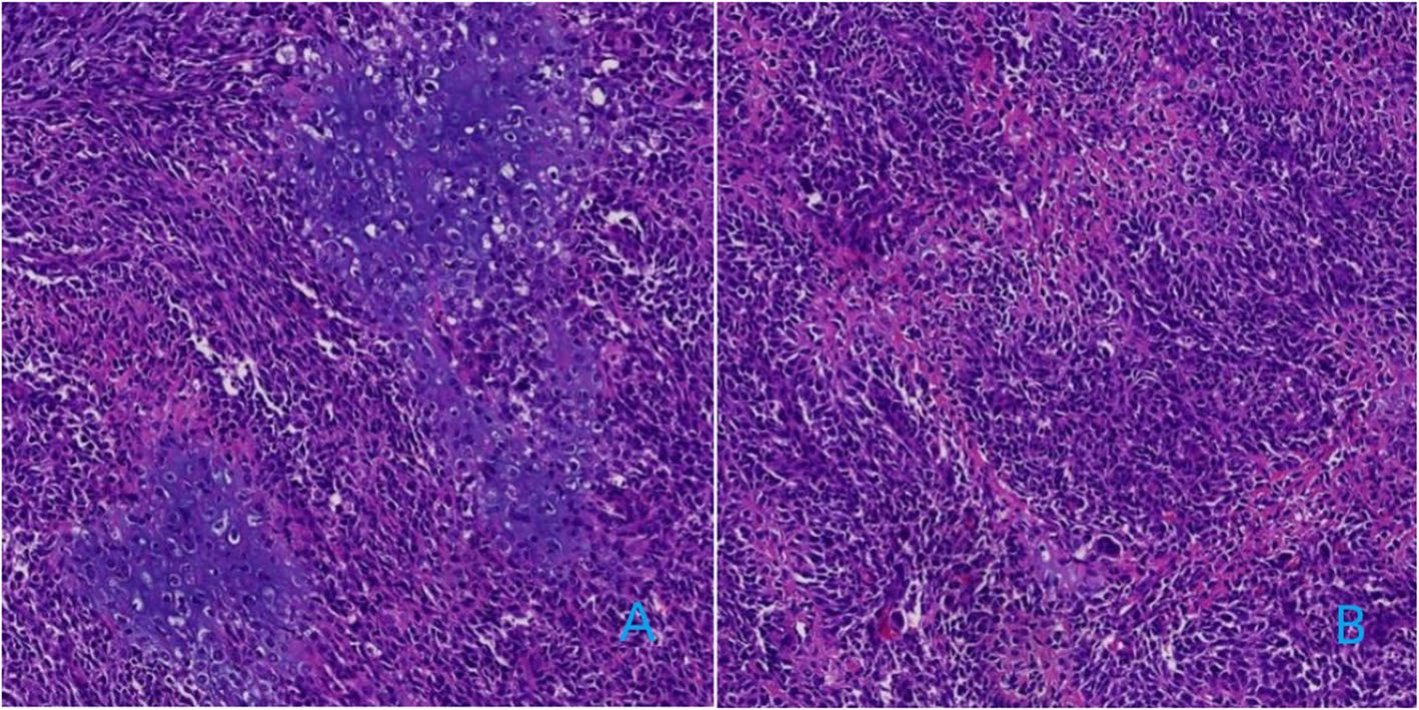
Extraskeletal osteosarcoma in the uterus. The irregularly distributed heteromorphic tumor cells were surrounded by osteoid matrix with fine branching lace-like structures (**B**), and cartilaginous tissue was seen in some areas (**A**)

Grossly, the tumor was a lobulated mass with well-defined or ill-defined borders, with foci of hemorrhage and necrosis, and a gray-white or gray-red cut surface. Microscopically, the tumor showed a wide range of highly atypical cells in random distribution, with variable proportions of spindle-shaped cells, osteoid tissue, and cartilaginous tissue. Various amounts of osteoid matrix and mineralization were observed around the tumor cells, forming a disorderly, fine branched lace-like structure (Fig. 7).

### Surgery, treatment, and tracking

This study included 11 patients, 8 of whom underwent wide excision, 1 of whom underwent marginal excision, and 2 of whom underwent excisional biopsy. Regarding surgical margins, 2 patients had positive margins, 3 had negative margins. Nine patients received chemotherapy after surgical resection, and one patient received both radiotherapy and chemotherapy (Table 3). Metastasis was found in 2 patients at the first diagnosis (cases 6 and 7); lung metastasis was found in patient 2 six months after surgery; and recurrence occurred in patient 4 two months after surgery. The follow-up period ranged from 2 to 54 months from diagnosis to death or loss of follow-up. Five patients survived after surgery (follow-up time ranged from 5 months to 54 months), and no obvious recurrence or metastasis was found by imaging examination. One patient who underwent local resection was alive with disease for 6 months. One patient died of lung metastasis after surgery (follow-up of 19 months), and 2 patients with excisional biopsy died of disease progression (follow-up of 8 months and 13 months). One patient died of respiratory failure 2 months after operation.

**Table 3:** Treatment and follow-up of extraosseous osteosarcoma

Case	Surgery	Chemotherapy	Relapses and transfer	Duration of follow-up(months)	The Outcome
Case1	Wide excision	Adriamycin	None	32	Survival
Case2	Marginal excision	Doxorubicin + methotrexate + ifosfamide + cisplatin	Lung metastases 6 months after surgery	19	death
Case3	Biopsy	/	None	11	death
Case4	Wide excision	Adriamycin + cisplatin	Relapse 2 months after surgery		
Case5	Wide excision	Doxorubicin + ifosfamide	None	12	Survival at last follow-up
Case6	Wide excision	Adriamycin + cisplatin	Extensive abdominal metastasis at first diagnosis	2	death
Case7	Wide excision	Adriamycin + cisplatin + methotrexate	First diagnosis metastasis	5	Survival
Case8	Wide excision	Isocyclophosphamide + cisplatin	None	54	Survival at last follow-up
Case9	Wide excision	/	None	34	Survival
Case10	Excisional biopsy	Adriamycin + methotrexate + ifosfamide + cisplatin	None	6	Survival with disease
Case11	Marginal excision	Adriamycin + cisplatin	None	13	death

/:untreated;—:loss of follow-up

## Discussion

EOS refers to osteosarcoma that occurs in organs and soft tissues outside bones. It is a rare soft tissue mesenchymal malignant tumour that was first reported by Wilson in 1941 [8] and is histologically similar to primary osteosarcoma of bone. The aetiology of EOS is still unclear. At present, many scholars accept the theory of chemical biology and believe that muscle fibroblasts are stimulated by external or internal factors, metaplastic osteoblasts or chondroblasts and evolve into osteosarcoma. Some studies have suggested that a history of trauma and irradiation may be related to the occurrence of EOS [1, 9, 10]. In the early stage, according to Lee et al. [9], the following 3 criteria must be met for a carcinoma to be identified as EOS: (1) it must occur in soft tissue without attachment to bone or periosteum, (2) have a uniform sarcomatous pattern(excluding mixed malignant stromal tumours), and (3) it must produce an osteoid and/or cartilage matrix. However, according to the latest WHO classification of tumours of bone and soft tissue, although EOS tumours do not originate from bone, they may involve bone structures with disease progression [11].

### Clinical manifestations

Unlike osteosarcoma, which originates in bone, EOS is more common in adults, especially in people over 50 years of age, and slightly more common in males [9, 12]. EOS occurs in the extremities, with the thigh being the most common site [3, 13], followed by the buttocks, upper limbs, trunk, abdominal cavity and retroperitoneum. EOS has also been reported in the lungs, liver, uterus, bladder and other parts [14–17]. The clinical features of EOS lack specificity. Generally, the onset is insidious, with no obvious symptoms in the early stage. The duration of the disease varies from several weeks to several decades. The most common clinical manifestation is a gradually increasing mass but can also present short-term rapid increase. The mass feels hard to the touch, and hardness is determined by the amount of bone tissue contained in the tumour. The mass is usually large, and lesions located in the abdominal cavity may cause symptoms such as abdominal pain and intestinal obstruction; a mass located in the limbs can limit the movement of adjacent joints.

### Imaging manifestations and histology

Although the imaging manifestations of EOS lack specificity, calcification and ossification are important manifestations. Calcification or ossification has been reported to occur in approximately 50% of cases of EOS [7]. X-ray of soft tissue masses is limited and is not good for soft tissue masses without calcification or bone tumour. Plain CT scans usually show soft tissue masses with uneven density, cystic degeneration and necrotic areas, accompanied by different degrees of calcification or ossification, the size and shape of which vary from nodular to mass. In this study, 2 patients had mature bone tumour masses. The distribution of calcification or the bone tumour is often heterogeneous, located in the centre and/or periphery of a mass; both situations were observed in the patients in this study, but most calcifications were located in the periphery, which is consistent with a previous report [2]. Histologically, EOS is mainly composed of spindle cells, bone or osteoid tissue, and cartilaginous tissue. The osteoid tissue of the tumour is directly formed by spindle cells and is mainly concentrated in the centre of the tumour, which is different from myositis ossificans [12]. Microscopically, the osteoid and osseous tissues are sometimes distributed in fine branching lace-like patterns and sometimes in broad sheets [4, 12].

On MRI, most of the masses had relatively clear boundaries, and some of them had complete or incomplete capsules.The capsule was composed of tumour cells, fibrous tissue, and various inflammatory components produced by the interaction between the tumour and surrounding normal tissues. On T1WI, the parenchyma of the mass was close to the muscle signal, and T2WI showed isointensity or slight hyperintensity; fibrous septa were evident in some masses, and both T1WI and T2WI showed low signals. Cystic degeneration is very common in EOS and can be clearly observed on MRI. In 2 patients in this study, haemorrhagic signals were observed in areas of cystic degeneration, manifesting as a fluid‒fluid sign. Haemosiderin, with a low signal on T2WI, was deposited in the lower layer of the cystic area. Excisional biopsy showed a grey‒red mass with multiple cysts on the cut surface in one case. This phenomenon has been reported for telangiectatic osteosarcoma [18–20]. Contrast-enhanced CT or MRI scans showed marked enhancement or mild enhancement in the parenchyma. MRI can also clearly show tumour invasion into surrounding structures.

### Differential diagnosis

EOS needs to be differentiated from a variety of soft tissue osteoblastic lesions, including myositis ossificans, dermatomyositis with ossification, and a variety of soft tissue tumours, such as extraskeletal chondrosarcoma, synovial sarcoma, undifferentiated pleomorphic sarcoma, fibrosarcoma, and liposarcoma.

Myositis ossificans is a nonneoplastic ectopic bone and cartilage entity formed locally near muscle tissue and bone. In the early stage of the disease, it is difficult to distinguish myositis ossificans from EOS. With the progression and maturation of the disease, the characteristic zoning phenomenon, well-organized mature layered bone in the periphery, intermediate osteoid area and central immature nonossifying cell (fibroblast) lesions are observed in histology and imaging [21]. On imaging, myositis ossificans showed progressive mineralization from the periphery to the centre with higher peripheral density than the centre, while the EOS showed reverse zoning (central deposition of bone-like material and peripheral proliferation of atypical spindle cells). Most patients with myositis ossificans have a history of trauma and are prone to paraplegia [21, 22]. Dermatomyositis with mineralization is more likely to occur in patients with juvenile myositis. Calcium deposits are found in the skin, subcutaneous tissue or deeper fascia and muscle. The morphology is diverse, usually diffuse, linear, flaky, and can gradually develop into a massive solid mass [23]. Notably, both myositis ossificans and dermatomyositis can be secondary to EOS.

The majority of soft tissue chondroma occurs in the finger region, followed by the foot, but occurrence in other parts is rare. The tumour cells of extraskeletal chondrosarcoma are surrounded by a cartilage matrix, and the peripheral cartilage matrix is mineralized [24, 25]. Hyaline cartilage nodules with a high water content and peripheral cartilage mineralization are characteristic findings. Imaging shows a typical “ring-arc” cartilage matrix mineralization, and the nonmineralized area has a high water content, low density on CT, and high signal intensity on MRI T2-weighted imaging [26]. However, these signs are less common in high-grade chondrosarcomas, showing more tumour cells, less cartilage matrix and less mineralized areas.

Osteoid or bone formation can be found in malignant tumours such as undifferentiated sarcoma, synovial sarcoma, and liposarcoma, and osteogenesis is mostly localized and can be reactive and neoplastic. Undifferentiated sarcoma has clinical and pathological similarities with EOS. It is difficult to distinguish EOS with less osteogenesis, which requires more adequate tissue sampling. Undifferentiated sarcoma does not have the disorderly, fine branching lace-like structure of EOS [12]. Clinically, undifferentiated/unclassified sarcoma and EOS are common in adult limbs, which are prone to cystic degeneration and bleeding, but undifferentiated/unclassified sarcomas have more fibrous components or fibrous septa, a low signal on T2WI, and progressive or continuous enhancement in parenchyma [27].

Synovial sarcoma can occur at any age. It is more common among young and middle-aged people and usually occurs near the large joints of the extremities [28, 29]. Synovial sarcoma manifests as “triple signal intensity” on T2WI, i.e., low signal, isointensity, and high signal area relative to fat, corresponding to fibrous tissue, haemorrhage, and cystic components [30]. Calcification is also a common sign [29]. Abdominal EOS mainly needs to be differentiated from gastrointestinal exophytic stromal tumours and omentum and mesenteric stromal tumours. This requires careful observation of the relationship between the mass and the adjacent bowel. A mass that is closely related to the bowel and can change its position should be considered a stromal tumour first.

### Treatment and survival

The prognosis of EOS is generally poor, and recurrence and metastasis are common, among which the lungs are the most common site for recurrence, followed by lymph nodes, liver, bone, soft tissue, etc. [7]. The 5-year overall survival rate in the current study ranged from 25–77% [31, 32]. In our study,Four patients died (follow-up time ranging from 2 to 49 months), and the survival rate was approximately 63%. Previous studies have shown that a tumour diameter less than 5 cm is associated with a better prognosis [1, 3]. Cases with masses less than 5 cm in diameter showed better prognosis in our study. At present, wide excision is the main treatment method, followed by postoperative adjuvant radiotherapy and chemotherapy. However, for elderly patients and abdominal mass surgery, caution should be taken. In this group, one patient with uterine osteosarcoma was admitted to the hospital due to dyspnoea one month after surgery and eventually died of respiratory failure. Recent studies have shown that the survival rate of patients with negative surgical margins is better [1, 3, 13]. Of these 8 patients who underwent surgery,2 patients had positive surgical margins. 1 patient had lung metastasis 6 months after operation and died 19 months after operation. Another patient declined postoperative radiation therapy and had a recurrence 2 months after surgery. The treatment effects of radiotherapy and chemotherapy for EOS are controversial. Several studies suggest that neither radiotherapy nor chemotherapy can improve the survival rate of patients with EOS but that adjuvant radiotherapy reduces the recurrence rate of patients with radically resectable EOS; however, chemotherapy cannot reduce the risk of systemic recurrence [3, 33, 34].

Several studies have shown that alkaline phosphatase and lactate dehydrogenase levels are correlated with the prognosis and metastasis of osteosarcoma [35–37]. High levels of alkaline phosphatase and lactate dehydrogenase were significantly associated with reduced overall survival (OS). In our study, of the 4 patients with elevated alkaline phosphatase levels, 2 died (2 months and 11 months of follow-up), and 1 survived with disease (5 months of follow-up). Of the 4 patients with normal alkaline phosphatase levels, 1 died after 19 months of follow-up, and the others survived without disease(12 months, 32 months and 34 months of follow-up).

Because this study was a retrospective study and due to different policies in different regions and different family conditions of patients, the management of examination, surgery, chemotherapy and follow-up were not consistent. Unfortunately, there were 3 patients with missing surgical margin information, which is very important for the treatment of patients. In addition, there are few cases in this group, and some cases are followed up for a short time, so more time is needed to observe.Regular follow-up and scientific management of patients with EOS are very important for a better understanding of EOS.

## Conclusions

In summary, the diagnosis of EOS requires a combination of clinical, imaging and histological examinations and needs to be differentiated from various tumours. Cystic degeneration and necrosis; mineralization is common, especially thick and lumpy mineralization are more meaningful for diagnosis. Haemorrhage within the mass may show signs of a liquid‒fluid level. Extended resection is still the first choice for localized lesions. For patients with positive surgical margins or metastases, adjuvant chemoradiotherapy is needed. Serum alkaline phosphatase and lactate dehydrogenase levels have certain significance in evaluating the survival of patients with EOS.

## Data Availability

Data to replicate findings are in the Figures and Tables of the main paper. Due to patient privacy protection, any additional materials of the study are only available upon individual request directed to the corresponding author.

## Abbreviations

EOS: extraskeletal osteosarcoma CT:computed tomography
MRI: magnetic resonance imaging DR:digital radiography
MPR: multiplanar reconstruction
